# Alcohol and substance use in extreme environment temperatures exposures, neural mechanisms, coping mechanisms, substance use disorders, and increased hospital visits

**DOI:** 10.3389/fnhum.2026.1677947

**Published:** 2026-02-05

**Authors:** Godfrey S. Bbosa, Haruna Muwonge

**Affiliations:** 1 Department of Pharmacology and Therapeutics, Makerere University College of Health Sciences, Kampala, Uganda; 2 Department of Physiology, Makerere University College of Health Sciences, Kampala, Uganda

**Keywords:** alcohol and substances, extreme environmental temperatures, hospitalization, mechanisms, mental illnesses, stress, substance use disorder (SUD)

## Abstract

Extreme environmental temperature disasters exposure pose a health challenge worldwide, including the use of alcohol and other substances, thus causing a risk of substance use disorders, and increased hospital visits. A review explored alcohol and other substance use in extreme environment temperature exposures, their mechanisms to substance use disorders and hospital visits. Relevant information used in the review was obtained through a literature search of different databases and a Google search using Boolean search with different search terms. Extreme environmental temperatures, especially in heat waves, leading to chronic stress and the development of mental disorders that trigger the use and abuse of alcohol and other drugs, especially alcohol, opioids, cocaine, cannabis, ecstasy, tobacco smoking, as coping mechanisms against heat stress–induced mental illnesses. Abuse of these substances often leads to SUD, drug intoxication, dehydration, and other health challenges often associated with increased hospital visits during the extreme weather temperatures (hot and cold). This conundrum of extreme weather, mental illnesses, and alcohol and substance use, pose significant public health challenges to the individual, communities, and society, and the already dilapidated healthcare systems, especially in developing nations. However, further studies are needed to understand the extreme weather temperatures, stress, mental illnesses, alcohol and other substances, and SUD interaction that can aid in developing intervention programs. Extreme weather temperature exposure triggers alcohol and other substance use, often leading to substance use disorders, hospital visits (hospitalization), and death, posing health a challenge to healthcare facilities, especially in developing nations.

## 1 Introduction

**Figure 1:**
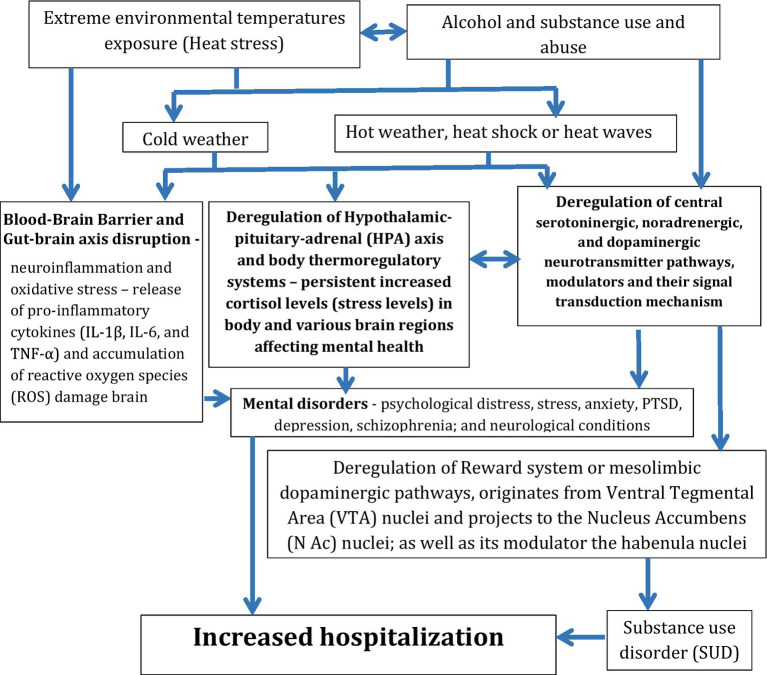
Extreme weather temperature exposure interaction with the HPA axis affecting various brain regions including reward center leading to mental disorders, alcohol and substance use disorder (SUD), and increased hospitalization.

Substance abuse and use have continued to be a serious global public health problem (Lange et al., 2024; UNODC, 2022; NIDA, 2025), due to a number of risk factor exposures, including extreme environmental temperatures often leading to death in the different parts of the world, genetic factors and history of chronic diseases like mental disorders and substance use (WHO, 2021, 2024; Mukerjee and Visser, 2025; Liu et al., 2021; Buguet et al., 2023). Extreme climate temperatures are increasingly recognized in chronic stress and the development of mental illnesses such as anxiety, depression, suicidal behaviors, post-traumatic stress disorder, sleep disorders, schizophrenia, sleep disorders, alcohol and substance use, substance use disorder (SUD), and increased hospital visits (WHO, 2021, 2024; Mukerjee and Visser, 2025; Liu et al., 2021; Buguet et al., 2023). Both hot and cold extreme weather conditions exposure affects various body physiological process (Figure 1) including (1) the hypothalamic–pituitary–adrenal (HPA) axis that modulates stress response, neurophysiological processes in the brain and sleep hygiene through cortisol and Dehydroepiandrosterone (DHEA) hormones (Buguet et al., 2023; WHO, 2024; Mbiydzenyuy and Qulu, 2024; Leistner and Menk, 2020; Dunlavey, 2018; Lõhmus, 2018); (2) various central neurotransmitters in the brain, their pathways and signaling cascades (Lõhmus, 2018; Garraway and Hochman, 2001; Lomax and Green, 1975); (3) body water hemostasis (dehydration), integrity of the blood–brain barrier (BBB) causing leakage of proteins, neurotoxins and ions in the brain (Kadry et al., 2020; Chou et al., 2025; Małkiewicz et al., 2019; Lee et al., 2025); (4) activation of the neuroimmunological responses and deregulation of heat shock proteins (HSP) (Buccellato et al., 2007; Dukay et al., 2019; Wang et al., 2023; Bardai et al., 2012; Chen et al., 2021; Nieto-Estevez et al., 2022) and deregulation gut-brain axis (Appleton, 2018); and (5) gene expression via the Histone Deacetylase 1 (HDAC1) epigenetic mechanisms (Buccellato et al., 2007; Dukay et al., 2019; Wang et al., 2023; Bardai et al., 2012; Chen et al., 2021; Nieto-Estevez et al., 2022). Deregulation of all these processes by extreme weather exposure leads to mental disorders, cognitive dysfunction, sleep disorders, and use of alcohol and other substances as coping mechanism often causing SUD, heat- and substance-induced dehydration and increased hospital visits (Figure 1) (Mukerjee and Visser, 2025; Liu et al., 2021; Buguet et al., 2023; WHO, 2024; Lõhmus, 2018). The extreme weather exposure has been reported to affect billions of people worldwide with 489,000 heat-related deaths annually recorded especially among the vulnerable people in low-middle income countries with poor heat intervention measures in place (WHO, 2021, 2025a).

### 1.1 Vulnerable population to extreme weather conditions leading to mental disorders, substance use and hospital visits

The extreme weather conditions and disasters has been reported to affect billions of workers worldwide especially among the vulnerable population such as in Southern Asia, sub Saharan and Western Africa (Buguet et al., 2023; WHO, 2013, 2024, 2025a; Butsch et al., 2023; Dodman et al., 2023; Minh et al., 2025; ILO, 2019; Rony and Alamgir, 2023; Hayes and Poland, 2018). The most reported vulnerable populations include individuals with existing mental disorders, those on certain psychiatric medications like antidepressants, antipsychotics, and substance use disorders that deregulate the body thermoregulatory system (Buguet et al., 2023; WHO, 2013, 2024, 2025a; Butsch et al., 2023; Dodman et al., 2023; Minh et al., 2025; ILO, 2019; Rony and Alamgir, 2023; Grant et al., 2025; Thompson, 2021; Hayes and Poland, 2018). Others include those individuals with SUD, chronic diseases such as cardiovascular diseases, respiratory diseases, diabetes mellitus, neurological diseases; children, homeless individuals with no shelter, older individuals, fast responders to disasters, pregnant women (Buguet et al., 2023; WHO, 2013, 2024, 2025a; Butsch et al., 2023; Dodman et al., 2023; Minh et al., 2025; ILO, 2019; Rony and Alamgir, 2023; Grant et al., 2025; Thompson, 2021; Hayes and Poland, 2018). Furthermore, those individuals residing in tropical and subtropical climates, outdoor workers such as construction workers, open market workers, manual workers on farms, casual laborers especially those who lack robust social protections, fisheries, low-income populations, those living in cities poorly planned, street vendors especially in low-middle income countries and motorbike drivers especially men gender (Buguet et al., 2023; WHO, 2013, 2024, 2025a; Butsch et al., 2023; Dodman et al., 2023; Minh et al., 2025; ILO, 2019; Rony and Alamgir, 2023; Hayes and Poland, 2018). A study among outdoor workers has reported a prevalence of heat-related ill symptoms to range from 64.0–90.3% (Rossi et al., 2025). These individuals suffers from chronic stress, mental disorders, sleep disorders, substance use leading to substance use disorder (SUD) or exacerbate it, dehydration, heat exhaustion and stroke and many others leading to hospitalization and often death (Liu et al., 2021; Buguet et al., 2023; WHO, 2024; ILO, 2019; Lawrance et al., 2022). As a way to address the heat stress and health challenges, the international Labor organization (ILO) and World Health Organization (WHO) and many others have provided guidance on protection of workers against extreme weather conditions especially among the vulnerable population (WHO, 2024; ILO, 2019). However, some individuals try to overcome these extreme environmental temperature-induced mental disorders by use of alcoholic beverages and other substances as a coping mechanism. *A substance is any chemical product that is naturally occurring, semi-synthesized, or synthesized and may be legal but controlled. or legal but prescribed with restrictions, or illegal with psychoactive properties that alter the perceptions, thoughts, awareness, emotions, consciousness, cognition, or mood and behaviors of the affected individual* (Lange et al., 2024; UNODC, 2022; NIDA, 2025). The enhanced substances used (Table 1) include (1) *central nervous system (CNS) stimulants* like cocaine, amphetamines, methamphetamines, caffeine, nicotine in tobacco products, and khat; (2) *narcotics* such as opioids like morphine, heroin, codeine, pethidine and many others; (3) *depressants* like alcohol, flunitrazepam (rohypnol), gamma hydroxybutyrate (GHB), diazepam, secobarbital and many others; (4) *hallucinogens* like lysergic acid diethylamide (LSD), mescaline, peyote, psilocybin, phencyclidine (PCP), N, N-Diisopropyl-5-methoxy-tryptamine (foxy), 3,4-Methylenedioxymethamphetamine (MDMA) or “ecstasy,” ketamine; *cannabinoids* like cannabis or marijuana; (5) *gases* like nitrous oxide or laughing gas that causes dissociation, euphoria, relaxation and hallucinogenic states of mind; (6) *inhalants* like gasoline such as jet fuel; amyl nitrate, glues, paint thinners, felt-tip marker fluid, dry cleaning fluids, hair spray, deodorants, spray paint, and (7) anabolic steroids like performance enhancing steroidal drugs (Lange et al., 2024; UNODC, 2022; NIDA, 2025). However, there is dearth of information on substances used in both extreme cold and hot weather conditions, effects on the body’s thermoregulatory processes, substance use disorder (SUD), and hospital visits. A narrative review was conducted to explore substance use in extreme weather temperatures as a coping mechanism for extreme environment-induced stress, anxiety, depression, and suicidal behaviors; effects on the body’s thermoregulatory processes, SUD, and hospital visits.

**Table 1:** Effects of extreme weather temperature on mental health, substance use disorders (SUD) and hospital visits.

Extreme weather temperature exposure	Substances use class and examples of commonly used	Effects on mental health and hospital visits	Ref
Heat stress or heat waves or heat shock and disasters	Alcohol beverages like beers, spirits, liquors, traditional brew and wines Central Nervous System (CNS) depressants like benzodiazepines such as diazepam, alprazolam, chlordiazepoxide, Flunitrazepam (Rohypnol) and gamma hydroxybutyrate (GBH) Antidepressant like sertraline paroxetine and fluoxetine CNS Stimulants like cocaine, “crack” cocaine, amphetamines, and methamphetamine (“crank”), and methylphenidate Tobacco and tobacco products, and nicotine Narcotic analgesics like opium, codeine, heroin, morphine, methadone and pethidine Cannabis like natural cannabinoids, marijuana and synthetics like dronabinol Hallucinogens like methylenedioxy-methamphetamine (MDMA) or Ecstasy Antipsychotic drugs like quetiapine, risperidone and olanzapine	Increased SUD and addiction Dehydration, heat exhaustion and, heat stroke (alcohol and opioids) Drug poisoning (alcohol) Opioid associated respiratory depression and death Substances exacerbates or lead to *anxiety, depression, posttraumatic stress disorder, and substance abuse disorders* Mental illnesses, SUD and poisoning lead to increased hospital visits	Liu et al., 2021; Lõhmus, 2018; Garraway and Hochman, 2001; Lawrance et al., 2022; Jamshidi and Dawson, 2019; Zhang et al., 2024; Trang et al., 2016; Berg, 2024; Luo and Huang, 2016; Vergunst et al., 2023; Tomassini et al., 2024; WHO, 2025b; Sinha, 2008; Beadle, 2023; Parks et al., 2023; Hensel et al., 2021; Cusack et al., 2011; Jane-Llopis and Matytsina, 2006; Spiers, 1995
Cold shock	Alcohol beverages abuse CNS Stimulants like amphetamine and cocaine Tobacco or cigarette smoking, and tobacco products and nicotine Narcotic analgesics like opium Cannabis like marijuana	Substances causes failure of thermoregulation leading to hypothermia, frostbite, Fatal drug overdoses and death Opioids associated respiratory depression and death. Some contrasting findings reported a protective effects of cold weather exposure	Spiljar et al., 2021; Kortelainen, 1987; Goedel et al., 2019; Anderton, 2019; Lewer et al., 2023; Freund et al., 1994; Hubbard and Pinelli, 2025; Ventura-Cots et al., 2019; Momperousse et al., 2007

## 2 Methodology

### 2.1 Search strategies

Literature search on the alcohol and substance use during extreme environment temperatures (cold and hot weather conditions), coping mechanisms during extreme heat stress, substance use disorders, and increased hospital visits were obtained from different databases including PubMed, Medline, Google scholar, Embase, Sage, World Health Organization website, Center for Disease Control and Prevention (CDC) website, Web of Science and Science Direct. Google Scholar, Science Direct, Web of Science, Medline and PubMed databases. The literature search was conducted between April to July 2025. Further relevant information was further identified using Google and Google scholar search involving Boolean search with different search terms including heat waves or heat shock or extreme environment heat exposure, cold shock or extreme environment cold exposure and alcohol or substances use and abuse or coping mechanism or body thermoregulatory mechanism or extreme environment-induced stress and anxiety or depression or suicidal behaviors or extreme environment-induced mental disorders or SUD and hospital visits. All retrieved articles were screened by the authors for relevance and the information was used in the review.

#### 2.1.1 Selection criteria of relevant articles

***Inclusion:*** All the articles included in the narrative review were from the authenticated scientific databases and the articles used had undergone the peer review before published. The articles used involved the effects of extreme weather exposure (cold and hot) on the body thermoregulatory mechanism leading to heat stress, and mental disorders such as anxiety, depression, and suicidal behaviors. This promote the use of alcohol and substance use as coping mechanism, and hence leading to substance use disorder and increased hospital visits. The articles used were from year 2000 to date.

***Exclusion:*** Articles earlier than 2000, and had not undergone the peer review process were excluded from the study.

## 3 Mechanisms of extreme heat exposure on mental health, substance use, and hospital visits

Various extreme heat exposure mechanisms including disruption of blood brain barrier (BBB) (Małkiewicz et al., 2019), activation of inflammatory processes in various regions of the brain, neuroendocrine system especially the HPA axis, and brain reward system have been implicated in mental disorders such as anxiety, mood disorders like depression and mania; schizophrenia, alcohol and substance use, substance use disorders (SUD) and hospital visits. These mechanisms can act as a combination of psychological, physiological such as stress hormones like cortisol and adrenaline through HPA axis deregulation, the brain neurotransmitter systems such as serotonin (5HT), dopamine (DA), norepinephrine (NA), neuromodulators and transcription factors like BDNF, all which are involved in mood regulation, thermoregulation and body temperature control (Figure 1) (Lõhmus, 2018; Garraway and Hochman, 2001; Lomax and Green, 1975; Volkow and Morales, 2015; Volkow et al., 2019). Furthermore, environment heat stress exposure affects gut-brain axis by causing gut microbiota dysbiosis, leaky gut causing inflammatory molecules like lithocholic acid from altered bile acid metabolism to enter blood circulation into the brain thus leading to neuroinflammation, reduced blood flow and oxygen in brain hence affecting the various neurotransmitters leading to mental illnesses, cognition and behavioral changes, and sleep disruption (Appleton, 2018). In addition, there are a number of drugs that affects the thermoregulation including antipsychotics, antiemetics, serotonin reuptake inhibitors (SSRI), monoamine oxidase inhibitors (MAOI), dextromethorphan, St John’s wort, antispasmodics, anticholinergic drugs, plant alkaloids like belladonna, Brugmansia, mushrooms like Amanita, phenthylamines like amphetamines, methamphetamines; cocaine, volatile anesthetics, depolarizing muscle relaxants like suxamethonium, tramadol, tapentadol, salicylates in overdose, dinitrophenol, antihistamines, diuretics, beta-blockers, stimulants and antibiotic linezolid, leading to acute drug-induced hyperthermia (Table 1) (Jamshidi and Dawson, 2019). Extreme heat exposure affects these mechanisms thus disrupting the body’s thermoregulation, sleep hygiene, blood brain battier (BBB) (Kadry et al., 2020; Chou et al., 2025; Małkiewicz et al., 2019; Lee et al., 2025), brain immune system (Buccellato et al., 2007; Dukay et al., 2019; Wang et al., 2023; Bardai et al., 2012; Chen et al., 2021; Nieto-Estevez et al., 2022), and cognitive function, and the individual coping mechanism (Mukerjee and Visser, 2025; Liu et al., 2021; Buguet et al., 2023; WHO, 2024; Lõhmus, 2018; Lomax and Green, 1975).

### 3.1 Effects of extreme weather temperature exposure on physiological body thermoregulation

Body thermoregulation controls the body internal temperature constant at approximately 37 °C or 98.6 °F even when the external environmental temperature changes (Mbiydzenyuy and Qulu, 2024; Leistner and Menk, 2020; Dunlavey, 2018; Lõhmus, 2018; Garraway and Hochman, 2001; Lomax and Green, 1975). The set temperature is vital for the enzymatic activities, body physiological processes including the metabolism and survival (Mbiydzenyuy and Qulu, 2024; Leistner and Menk, 2020; Dunlavey, 2018; Lõhmus, 2018; Garraway and Hochman, 2001; Lomax and Green, 1975). The thermoregulation is modulated by the hypothalamus in the brain, which work as the body’s thermostat and acts via the HPA axis, and it responds on the prevailing environmental temperatures. The deregulation of thermoregulation affects various neurotransmitters systems in the brain including the serotoninergic, dopaminergic, and noradrenaergic systems thus affecting the behavioral and mental states (Lõhmus, 2018; Garraway and Hochman, 2001; Lomax and Green, 1975). During extreme cold weather, the HPA axis acts in a way to conserve body heat through vasoconstriction, shivering, and piloerection (Goosebumps); while in extreme heat waves or stress, there is vasodilation, sweating and behavioral changes that seek for cold water, cool environment and removal of clothes as way to cool the body (Figure 1) (Mbiydzenyuy and Qulu, 2024; Leistner and Menk, 2020; Dunlavey, 2018; Lõhmus, 2018; Garraway and Hochman, 2001; Lomax and Green, 1975). In addition the hypothalamic–pituitary–adrenal (HPA) axis modulates body temperature (Mbiydzenyuy and Qulu, 2024; Leistner and Menk, 2020; Dunlavey, 2018; Lõhmus, 2018; Garraway and Hochman, 2001; Lomax and Green, 1975), regulation of digestion and metabolism and the sympathetic component of the autonomic nervous system (ANS) responsible for the fight-or-flight response, immune responses, and in the brain, it modulates the maintenance of mental health, mood, and metabolic energy levels (Mbiydzenyuy and Qulu, 2024; Leistner and Menk, 2020; Dunlavey, 2018; Lõhmus, 2018; Garraway and Hochman, 2001; Lomax and Green, 1975), and modulation of neuroinflammation (Spiljar et al., 2021). However, sustained extreme weather conditions exposure causes the deregulation of the HPA axis and the brain, physiological processes leading to mental disorders, sleep disorders and the use of alcohol and other substances as a coping mechanism that in turn leads to SUD and increased hospital visits (Figure 1) (Zhang et al., 2024; Trang et al., 2016).

### 3.2 Effects of extreme weather exposure on blood brain barrier and neuroimmune system

Exposure to the extreme weather conditions especially heat shock (severe heat exposure or heatstroke) causes the disruption of the tight junctions of the blood–brain barrier (BBB) by reducing the claudin-5, ZO-1, and occludin proteins that seal BBB thus increasing the brain permeability and the leakage of proteins, neurotoxins, ions such as calcium and pathogens from blood into the brain (Kadry et al., 2020; Chou et al., 2025; Małkiewicz et al., 2019; Lee et al., 2025). These leaked substances in the brain causes neuroinflammation, peripheral immune cell infiltration such as peripheral immune cells (monocytes and granulocytes), and the resident microglial and astrocyte activation leading to neurotoxicity of neurons and endothelial cells further causing BBB breakdown (Kadry et al., 2020; Chou et al., 2025; Małkiewicz et al., 2019; Lee et al., 2025). The microglial activation shifts from the protective state to a destructive state, activation of pro-inflammatory proteins such as CD80/86 that activates cytokines mainly Tumor Necrosis Factor alpha (TNF-*α*) and Interleukin-1-beta (IL-1β), and elevated anti-inflammatory proteins like Cluster of Differentiation 206/163 (CD206/163) that damage the brain, production of oxidative stress especially reactive oxygen species (ROS), and matrix metalloproteinases (MMPs) that further worsen the cellular damage in brain, neuronal death or apoptosis, disrupt cerebral blood flow and edema formation (Kadry et al., 2020; Chou et al., 2025; Małkiewicz et al., 2019; Lee et al., 2025). In addition, heat shock disrupts the brain heat shock proteins (HSPs) such as HSP27, HSP60, HSP70 and HSP90 that protects neurons from stress such as heat stress (Leistner and Menk, 2020; Dukay et al., 2019), traumatic brain injury, ischemia, and neurodegeneration by maintaining the protein homeostasis, modulation of neuroinflammation, promote neuronal survival, eliminating the misfolded proteins, and preventing aggregated proteins associated with diseases like Alzheimer’s and Huntington’s disease (Lee et al., 2025; Wang et al., 2023; Bardai et al., 2012; Chen et al., 2021; Nieto-Estevez et al., 2022). Heat shock has also been reported to influence the epigenetic mechanisms in the brain such as Histone Deacetylase 1(HDAC1) that plays a vital role in gene expression and protein interactions in the brain including neuronal development, differentiation, apoptosis and survival thus acting as a “molecular switch,” and its damage is implicated in a number of brain diseases such as stroke, Traumatic Brain Injury (TBI), Alzheimer’s, parkinson’s disease, cognitive dysfunction and mental disorders (Wang et al., 2023; Bardai et al., 2012; Chen et al., 2021; Nieto-Estevez et al., 2022). These changes affects various central neurotransmitter system such as the excitatory glutaminergic system, inhibitory gabargic system and the neuromodulator dopaminergic pathways, noradrenergic system and serotoninergic system (5-HT) and their signaling pathways leading to brain dysfunction and mental and neurological disorders (Figure 1) (Lõhmus, 2018; Garraway and Hochman, 2001; Lomax and Green, 1975; Volkow and Morales, 2015; Volkow et al., 2019). The highlighted mental disorders include PTSD, anxiety, depression, suicidal behaviors, decline in the cognitive function, neurological disorders like dementia, heat stroke, seizures, fatigue, migraines, sleep disorders, Parkinson’s disease and Alzheimer’s disease (Lõhmus, 2018; Garraway and Hochman, 2001; Lomax and Green, 1975; Volkow and Morales, 2015; Volkow et al., 2019). Similarly, extreme cold exposure also leads to neuroinflammation thus leading to reduced brain activity and behaviors, and mental illnesses (Kadry et al., 2020; Chou et al., 2025; Małkiewicz et al., 2019; Lee et al., 2025; Wang et al., 2023; Bardai et al., 2012; Chen et al., 2021; Nieto-Estevez et al., 2022). However, some reports show that cold exposure has protective effects against brain neuroinflammation via immunologic reprogramming (Spiljar et al., 2021).

### 3.3 Role of heat stress on brain neurotransmitter system, behavioral processes and mental illnesses

Similar to the body’s responses to cold stress and mental illnesses, extreme heat stress exposure causes increased release of cortisol in the body via the prolonged HPA axis activation (WHO, 2024; Mbiydzenyuy and Qulu, 2024; Leistner and Menk, 2020; Dunlavey, 2018; Lõhmus, 2018; Garraway and Hochman, 2001; Lomax and Green, 1975). The high cortisol levels cause the deregulation of the various brain regions and their associated neurotransmitter pathways and their function, including the serotoninergic system (5-HT) that modulate mood; noradrenergic system (NE) that modulate mood and arousal, and the dopaminergic pathways that modulate the reward and motivation via the brain reward system (Lõhmus, 2018; Garraway and Hochman, 2001; Lomax and Green, 1975). The disruption of the reward pathway leads to SUD, addiction, and increased hospital visits, as well as the development of other mental illnesses (Zhang et al., 2024; Trang et al., 2016). Additionally, exposure to excessive heat may drive stress through the deregulation of the many brain neurochemicals and their associated signal transduction mechanisms (Figure 1) including (1) Brain-derived neurotrophic factor (BDNF)/Extracellular Signal-Regulated Kinase ½ (ERK1/2)/ cAMP response element-binding protein (CREB) (BDNF/ERK1/2/CREB) axis that modulates neuronal survival and plasticity; (2) p38-mitogen-activated protein kinases (MAPKs; p38-MAPK) pathway that modulates the tissue-specific pro- or anti-apoptotic events in the brain; (3) heat shock proteins (HSPs) (Leistner and Menk, 2020; Dukay et al., 2019), and its protective effects thus increase apoptosis through calcium dyshomeostasis via the Protein kinase-like endoplasmic reticulum kinase (PERK)/ Phosphorylation of eukaryotic initiation factor-2α (eIF2α)/C/EBP homologous protein (CHOP; p-PERK/p-eIF2α/CHOP) signaling pathways (Chauhan et al., 2021; Kefaloyianni et al., 2005). In addition, excessive heat affects the gut-liver-brain axis (GLBA) which also modulates excessive heat, and its deregulation leads to mental and mood disorders, especially anxiety and depressive illnesses (Figure 1) (Roy et al., 2024; Toader et al., 2024; Yan M. et al., 2023). Notably, heat stress exposure also further disrupts the blood–brain barrier (BBB), the protective layer of the brain, and hence causes the dangerous chemical substances to leak into the brain parenchyma, thus affecting its function through inflammatory processes (Kiyatkin and Sharma, 2009; Yan Z. et al., 2023). This further may lead to mental illnesses such as anxiety, depressive illnesses, suicidal behaviors, sleep disorders, sleep disorders and cognitive dysfunction (Liu et al., 2021; Buguet et al., 2023; WHO, 2024; ILO, 2019; Lawrance et al., 2022). In addition, heat stress exposure causes inflammatory reactions, which may further trigger or worsen the mental illnesses in vulnerable individuals, and increased hospital visits (Mukerjee and Visser, 2025; Liu et al., 2021; Buguet et al., 2023; WHO, 2024; Lõhmus, 2018).

To cope with extreme weather heat or cold temperature exposure, practicing healthy coping behaviors like healthy diet consumption, continuous hydration, daily physical activity, good sleep hygiene, engaging in stress-reducing activities like relaxation techniques and mindfulness, maintaining social interactions, and practicing behavior mechanisms aimed at cooling the body during exposure to extreme heat or warming the body during exposure to extreme cold stress conditions is vital (Yahiro et al., 2023; Mota-Rojas et al., 2021).

However, some individuals engage in unhealthy behaviors that involve the use and abuse of alcohol and other substances that target the brain reward system as a coping mechanism against heat stress that may be detrimental to the body and the brain, thus triggering mental illness development, alcohol and other substance use, SUD, and addiction or their exacerbation (Liu et al., 2021; Buguet et al., 2023; WHO, 2024; ILO, 2019; Lawrance et al., 2022), thus increasing the hospital visits noted during the period of extreme hot weather temperatures experienced during various disasters (Rony and Alamgir, 2023; Trang et al., 2016; Barlattani et al., 2024).

### 3.4 Cold weather temperatures exposure in mental illnesses

Extreme cold weather temperatures exposure make the body send signals to the hypothalamus, leading to activation of the thermoregulatory system through the release of cortisol from the adrenal gland (Mbiydzenyuy and Qulu, 2024; Yankouskaya et al., 2023; Garraway and Hochman, 2001; López-Ojeda et al., 2024) and activation of norepinephrine components from the sympathetic nervous system (SNS) to influence body heat production and shivering (Mbiydzenyuy and Qulu, 2024; Yankouskaya et al., 2023; Garraway and Hochman, 2001; López-Ojeda et al., 2024). In addition, cortisol in the brain causes a reduction in the brain-derived neurotrophic factor (BDNF) levels in the various brain regions like the prefrontal cortex and striatum (Mbiydzenyuy and Qulu, 2024; Yankouskaya et al., 2023; Garraway and Hochman, 2001; López-Ojeda et al., 2024); brain volume reduction, especially in the hippocampus, causing cognitive deficiencies and emotional states, neurodegenerative disorders, and increased inflammatory reactions; and disruption of various brain neurotransmitter pathways, including serotoninergic, noradrenergic, and dopaminergic pathways, leading to the development of anxiety, depressive illnesses, and suicidal behaviors (Mukerjee and Visser, 2025; Liu et al., 2021; Zhang et al., 2024; Trang et al., 2016), of which these are exacerbated by substance use and hence the development of SUD and increased hospital visits (Rony and Alamgir, 2023; Trang et al., 2016; Barlattani et al., 2024).

## 4 Brain reward system and alcohol and substance use during extreme weather temperature exposures

The brain reward system or the mesolimbic dopaminergic pathway modulates a number of brain behaviors such as motivation, emotions, learning and memory, sleep hygiene, reinforces behaviors by responding to natural reward stimuli as well as alcohol and substances and addictive behaviors (Volkow and Morales, 2015; Volkow et al., 2019; Berg, 2024; Lewis et al., 2021). The pathway also modulates many other neurotransmitter pathways like the noradrenergic, serotonergic, and dopaminergic systems in the brain, thus influencing motivation and reward, emotion processing, pain, sleep cycle and circadian rhythms, and behavioral adaptation (Volkow and Morales, 2015; Volkow et al., 2019; Berg, 2024; Lewis et al., 2021; Piper et al., 2024; Luo and Huang, 2016; Vergunst et al., 2023; Tomassini et al., 2024; WHO, 2025b; Sinha, 2008). The dysfunction in the reward system and habenula due to environmental insults like extreme weather temperature exposures, alcohol, and other substances (artificial rewards) alters their functions, leading to mental illnesses, alcohol and other substance use, SUD, intoxication, and addiction behaviors (Yankouskaya et al., 2023; Berg, 2024; Lewis et al., 2021; Piper et al., 2024); that eventually lead to mental disorders, SUD and increased hospital visits.

### 4.1 Interaction of extreme weather temperature exposure and substance use

Exposure to climate change-driven extremes, including heat waves or extreme coldness in many countries worldwide including in Africa (Rony and Alamgir, 2023; WHO, 2025b; Sinha, 2008; Beadle, 2023; Parks et al., 2023; Hensel et al., 2021; Cusack et al., 2011; Jane-Llopis and Matytsina, 2006; Spiers, 1995), has been reported to influence and increase or exacerbate the burden of alcohol and other substance use and abuse, often leading to SUD, intoxication, and dehydration, especially with alcohol and opioids like heroin; cocaine, and cannabis, especially among the youth (Rony and Alamgir, 2023; WHO, 2025b; Sinha, 2008; Beadle, 2023; Parks et al., 2023; Hensel et al., 2021; Cusack et al., 2011; Jane-Llopis and Matytsina, 2006; Spiers, 1995). However, further research is needed to understand the interaction and effects of extreme weather temperature exposure, mental illness pathophysiology, and alcohol and substance use and abuse that would inform policy (Rony and Alamgir, 2023; WHO, 2025b; Sinha, 2008; Beadle, 2023; Parks et al., 2023; Hensel et al., 2021; Cusack et al., 2011; Jane-Llopis and Matytsina, 2006; Spiers, 1995). In addition, exposure to extreme weather temperatures disrupts individual daily life at the societal and community levels, thus affecting the body’s physiological processes and socioeconomic activities of the communities (Rony and Alamgir, 2023). Furthermore, it is reported that individuals cope with extreme weather temperature-induced stress and mental illnesses by using alcohol and other substances of abuse, especially among vulnerable individuals (Rony and Alamgir, 2023; WHO, 2025b; Sinha, 2008; Beadle, 2023). In addition, extreme temperature exposure may interact with other existing factors like genetics or family history, prior chronic stress, past experience of alcohol and substance use, health challenges, age, nature of occupation, geographical location, and many others to trigger or exacerbate mental illnesses, including anxiety disorders, cognitive dysfunction and memory, trauma, stressor-related disorders like post-traumatic stress disorder (PTSD), depressive illnesses, suicidal behaviors, neurological diseases, chronic and psychosocial stress, sleep disturbances, SUD, drug intoxication, increased hospital visits, and death due to substance abuse as a coping mechanism (Rony and Alamgir, 2023; Berg, 2024; Luo and Huang, 2016; Vergunst et al., 2023; Tomassini et al., 2024; WHO, 2025b; Sinha, 2008; Beadle, 2023; Parks et al., 2023; Hensel et al., 2021; Cusack et al., 2011; Jane-Llopis and Matytsina, 2006; Spiers, 1995).

## 5 Alcohol and substance use as a coping mechanism for extreme weather temperature exposure induced-mental illnesses

The abuse of various alcoholic beverages and other substances (drugs) has continued to be a serious health problem worldwide, with detrimental health and mental effects and often leading to SUD and increased hospital visits that account for 3.3% of the global burden of diseases, with 16.8 million deaths annually (WHO, 2021, 2025b; Piper et al., 2024; Berg, 2024; Luo and Huang, 2016; Vergunst et al., 2023; Tomassini et al., 2024; Sinha, 2008; Beadle, 2023; Parks et al., 2023; Hensel et al., 2021; Cusack et al., 2011; Jane-Llopis and Matytsina, 2006; Spiers, 1995). Substance use involves the harmful use or hazardous use of psychoactive substances, which may be legal or illegal, including alcohol, tobacco products, recreational drugs like cannabis, cocaine, heroin, lysergic acid diethylamide (LSD), and khat; inhalants; and prescription medications such as opioids like morphine and pethidine; benzodiazepines; and barbiturates (WHO, 2021, 2025b; Berg, 2024; Luo and Huang, 2016; Vergunst et al., 2023; Tomassini et al., 2024; Sinha, 2008; Beadle, 2023; Parks et al., 2023; Hensel et al., 2021; Cusack et al., 2011; Jane-Llopis and Matytsina, 2006; Spiers, 1995). These substances reach the body by chewing, injections, and inhalation. In the body, these drugs alter the individual’s behaviors as a coping mechanism to extreme cold and hot weather as well as the ability to rehydrate, seeking shelter from extreme heat or putting on warm clothes in response to extreme cold exposures (Luo and Huang, 2016; Vergunst et al., 2023; Tomassini et al., 2024; WHO, 2025b; Sinha, 2008; Beadle, 2023; Parks et al., 2023; Hensel et al., 2021; Cusack et al., 2011; Jane-Llopis and Matytsina, 2006; Spiers, 1995); however, drug intoxication and dehydration during extreme weather temperatures occur leading to increased hospital visits and often death.

Furthermore, the use and abuse of alcoholic beverages and other substances are triggered or exacerbated by the interaction of multiple factors, including genetic vulnerability; environmental stressors like extreme cold and heat exposure; social pressures; individual characteristics; health challenges; and existing mental illnesses like anxiety, depression, suicidal behaviors, post-traumatic stress disorder (PTSD), attention-deficit hyperactivity disorder (ADHD), bipolar disorder, personality disorders, and schizophrenia; and also neurological disorders, among others (Berg, 2024; Luo and Huang, 2016; Vergunst et al., 2023; Tomassini et al., 2024; WHO, 2025b; Sinha, 2008; Beadle, 2023; Parks et al., 2023; Hensel et al., 2021; Cusack et al., 2011; Jane-Llopis and Matytsina, 2006; Spiers, 1995). Exposure to extreme heat causes deregulation of the corticotropin-releasing factor (CRF) in the hypothalamic–pituitary–adrenal (CRF/HPA) axis, the extrahypothalamic CRF system that comprises of CRF neurons and systems found outside the hypothalamus mainly targeting the amygdala, the ANS arousal, and the central noradrenergic systems affecting motivation, learning, and adaptive systems like reward (Berg, 2024; Luo and Huang, 2016; Vergunst et al., 2023; Tomassini et al., 2024; WHO, 2025b; Sinha, 2008; Beadle, 2023; Parks et al., 2023; Hensel et al., 2021; Cusack et al., 2011; Jane-Llopis and Matytsina, 2006; Spiers, 1995). Chronic stress exposure and high cortisol levels in the brain during extreme cold and heat exposure, alter these systems, like the dopaminergic mesolimbic (reward) pathway, leading to reduced synthesis and release of dopamine in NAc, thus exposing the individuals to drugs of abuse, risk of addiction, SUD, and addictive behaviors and increased hospital visits. Furthermore, other neurotransmitter pathways affected include gabaergic, glutamatergic and cholinergic pathways that are also involved in modulation of the stress-associated risk of addiction to substances of abuse and addictive behaviors as well as emotions, motivation, learning, and adaptive systems (Berg, 2024; Luo and Huang, 2016; Vergunst et al., 2023; Tomassini et al., 2024; WHO, 2025b; Sinha, 2008; Beadle, 2023; Parks et al., 2023; Hensel et al., 2021; Cusack et al., 2011; Jane-Llopis and Matytsina, 2006; Spiers, 1995). In addition, cold shock activates the natural opioid neuropeptide brain neurotransmitter and neuromodulator, like the endorphins that act as beneficial body’s natural painkillers and mood boosters in response to stress (Berg, 2024; Luo and Huang, 2016; Vergunst et al., 2023; Tomassini et al., 2024; WHO, 2025b; Sinha, 2008; Beadle, 2023; Parks et al., 2023; Hensel et al., 2021; Cusack et al., 2011; Jane-Llopis and Matytsina, 2006; Spiers, 1995). Alcohol and other substances, affects perception of pain, and pleasurable activities like exercise, sex, eating, and laughter, as well as reducing the withdrawal symptoms and cravings in addiction to drugs and addictive behaviors, thus exposing them to SUD and hospital visits (Table 1) (Berg, 2024; Luo and Huang, 2016; Vergunst et al., 2023; Tomassini et al., 2024; WHO, 2025b; Sinha, 2008; Beadle, 2023; Parks et al., 2023; Hensel et al., 2021; Cusack et al., 2011; Jane-Llopis and Matytsina, 2006; Spiers, 1995).

### 5.1 Substances commonly used in extreme cold weather conditions

During exposure to extreme cold weather temperatures, different forms of alcoholic beverages; opioid pain relievers like morphine, pethidine, heroin; cocaine, cannabis (marijuana); 3,4-methylenedioxymethamphetamine (MDMA or ecstasy or molly), amphetamines, methamphetamine; cigarettes and tobacco products; khat use (Table 1), especially in East Africa, Yemen, and Southern Saudi Arabia; prescription and over-the-counter (OTC) medicines and many others are used (Berg, 2024; Luo and Huang, 2016; Vergunst et al., 2023; Tomassini et al., 2024; WHO, 2025b; Sinha, 2008; Beadle, 2023; Parks et al., 2023; Hensel et al., 2021; Cusack et al., 2011; Jane-Llopis and Matytsina, 2006; Spiers, 1995). These substances, especially amphetamines act as stimulants and thermogenic agents, acting directly or indirectly to activate the noradrenergic sympathetic nervous system (SNS) pathways and thyroid hormones from thyroid gland triggered by cold weather, thus causing the mobilization of body fats from adipose tissues and activation of mitochondrial proteins to up regulates the metabolic processes in the body, thus leading to increased body temperature (Lõhmus, 2018; Piper et al., 2024; Luo and Huang, 2016; Vergunst et al., 2023; Tomassini et al., 2024; WHO, 2025b; Sinha, 2008; Beadle, 2023; Parks et al., 2023; Hensel et al., 2021; Cusack et al., 2011; Jane-Llopis and Matytsina, 2006; Spiers, 1995). And at the same time, these agents cause vasoconstriction through activation of the α1-adrenoreceptor in the peripheral sympathetic nervous system, thus reducing body heat loss. Similarly, as these substances trigger heat production, they also cause dopaminergic synaptic plasticity in the VTA nuclei in the reward pathway, thus enhancing the drug-seeking behaviors or addiction or exacerbating it (Berg, 2024; Luo and Huang, 2016; Vergunst et al., 2023; Tomassini et al., 2024; WHO, 2025b; Sinha, 2008; Beadle, 2023; Parks et al., 2023; Hensel et al., 2021; Cusack et al., 2011; Jane-Llopis and Matytsina, 2006; Spiers, 1995). Furthermore, amphetamines, methamphetamines, and MDMA activate the dopamine transporters (DAT) in the nucleus accumbens (NAc), thus further strengthening the drug abuse seeking behavior and their use (Berg, 2024; Luo and Huang, 2016; Vergunst et al., 2023; Tomassini et al., 2024; WHO, 2025b; Sinha, 2008; Beadle, 2023; Parks et al., 2023; Hensel et al., 2021; Cusack et al., 2011; Jane-Llopis and Matytsina, 2006; Spiers, 1995). These findings underscore the effects of exposure to extreme environmental temperatures due to weather changes and their role in mental illnesses, use of alcohol and other drugs as a coping mechanism for weather changes, often leading to drug intoxication, dehydration, SUD, and increased hospital visits, and some cases of death. Thus, there is a need to create awareness and preventative measures to overcome these extreme weather temperature challenges and their impact on health.

### 5.2 Substances commonly used in extreme hot or heat waves and cold weather conditions

Similar to exposure to extreme cold weather, substance use and abuse presents a significant public health challenge globally during extreme heat events, resulting in SUD, drug intoxication, and dehydration, especially with alcohol, opioids, cocaine, and cannabis, leading to increased hospital visits or death (Table 1). Among substances used during extreme hot environments or heat waves, and coldness include different alcoholic beverages, cannabis, different forms of cocaine, amphetamine, methamphetamine, MDMA or ecstasy, opioids, heroin, gamma-hydroxybutyrate (GHB), ketamine, phencyclidine or phenylcyclohexyl piperidine (PCP), and psychotropic prescription drugs like antipsychotics, antidepressants, hypnotics, and anxiolytics (Table 1) (Berg, 2024; Luo and Huang, 2016; Vergunst et al., 2023; Tomassini et al., 2024; WHO, 2025b; Sinha, 2008; Beadle, 2023; Parks et al., 2023; Hensel et al., 2021; Cusack et al., 2011; Jane-Llopis and Matytsina, 2006; Spiers, 1995).

Extreme heat exposure and heat waves cause body dehydration, thus reducing the body water in which the alcohol and other substances can be diluted as well as reducing their elimination from the body through the kidneys and sweating, thus causing alcohol and other drug toxicity or poisoning (Table 1) (Beadle, 2023; Parks et al., 2023). During these periods of extreme heat waves, significant hospital visits and admissions due to alcohol and other substance abuse, SUD and their related effects like accidents (Table 1) and crimes have been reported to be on the increase worldwide, thus further providing evidence of increased substance use during the extreme weather conditions (Berg, 2024; Luo and Huang, 2016; Vergunst et al., 2023; Tomassini et al., 2024; WHO, 2025b; Sinha, 2008; Beadle, 2023; Parks et al., 2023; Hensel et al., 2021; Cusack et al., 2011; Jane-Llopis and Matytsina, 2006; Spiers, 1995).

Furthermore, studies have reported increased alcohol consumption and other substance abuse, especially stimulants and opioid abuse have been reported to increase hospital visits during heat waves (Rony and Alamgir, 2023; Trang et al., 2016; Barlattani et al., 2024; Beadle, 2023; Parks et al., 2023). These drugs interact with heat to cause deregulation of the thermogenic systems in the body through the HPA axis (Hensel et al., 2021; Spiers, 1995). These further exacerbate heat generation or loss in addition to the already exposed high environmental heat, leading to risk for heat-related illnesses like heat exhaustion or heatstroke, sleep deprivation, stress, and mental illnesses like anxiety, depression, suicidal behaviors, and exacerbation of further alcohol and substance abuse as a coping mechanism (Hensel et al., 2021; Spiers, 1995). Alcohol and nicotine use has been reported to cause deregulation of the thermogenic systems (Hensel et al., 2021; Spiers, 1995). In addition, extreme heat exposure disrupts the blood–brain barrier (BBB), thus causing permeability of dangerous chemical substances into the brain parenchyma, causing inflammation and brain cell damage that disrupts various central neurotransmitter systems and their signaling pathways, leading to heat stroke, cognitive impairment, sleep disorders, mental illnesses like anxiety, depression, and suicidal behaviors; neurological disorders; further alcohol and substance use (Yankouskaya et al., 2023; Berg, 2024; Lewis et al., 2021; Piper et al., 2024); and SUD, intoxication, and increased hospital visits (Table 1). Like in extreme coldness, findings highlight the role of increased weather heat exposure in the development or exacerbation of mental illness and the use of alcohol and other drugs as coping mechanisms, thus worsening SUD, drug intoxication, dehydration, and increased hospital visits (Table 1) (Rony and Alamgir, 2023; Kortelainen, 1987; Goedel et al., 2019; Anderton, 2019; Lewer et al., 2023; Freund et al., 1994; Hubbard and Pinelli, 2025; Ventura-Cots et al., 2019; Momperousse et al., 2007), creating a burden to healthcare facilities; even cases of death occur.

## 6 Effects of hot climate exposure, mental illnesses, SUD and hospital visits

Alcohol and other drugs are commonly used and abused during extreme weather temperatures (cold and hot) as a measure to overcome heat challenges or as a stress coping mechanism, thus often leading to increased hospital visits due to overdose, excessive generation of body temperature (hyperthermia) often causing heat stroke, substance use disorder (SUD), addiction, and death, especially respiratory failure due to drug overdose (Tomassini et al., 2024; WHO, 2025b; Sinha, 2008; Beadle, 2023; Parks et al., 2023; Hensel et al., 2021; Cusack et al., 2011; Jane-Llopis and Matytsina, 2006). Studies have reported that exposures to extreme weather conditions such as heat waves, heat stroke and cold shock of frostbite leads to both short- and long-term health crises such as chronic stress, mental disorders such as schizophrenia, mood disorders, neurotic disorders, and SUD thus requiring hospital admissions (Liu et al., 2021; Lawrance et al., 2022; Zhang et al., 2024; Trang et al., 2016). However, many developing countries have established strategies or interventions to address the effects of extreme weather exposure to vulnerable population and thus reduce its effects and hospital admissions, but low-income countries lack such interventions (Rony and Alamgir, 2023; Lawrance et al., 2022; Baecker et al., 2025; Stewart-Ruano et al., 2025). Furthermore, studies are needed, especially on the effects of extreme cold weather on alcohol and other substances use and abuse, and the hospital visits. Understanding the extreme environmental temperature disaster exposure, and its role in the pathophysiology of mental illnesses, and substances use interaction, often leading to SUD, addiction, drug intoxication; increased hospital visits, and even the associated death, is vital in implementing preventative measures during the disaster periods (Stewart-Ruano et al., 2025; Mosel and Newman, 2025; Hayes and Poland, 2018; WHO, 2013; Lennox and Cecchini, 2008).

## 7 Limitation of the review

The present review is limited by being a narrative review and therefore a systematic review will be considered. In addition, this is a new emerging area in the awake of climate change and its impact on health, mental health, SUD and hospital visits, and therefore limited research has been conducted on the subject area.

## 8 Strengths of the review

The study is highlighting the role of extreme weather temperatures on the body thermoregulatory mechanisms, which in turn deregulates a number of physiological processes in the body including the brain such as the HPA axis, BBB, neurotransmitter system, neuroimmunological responses thus triggering the developmental of mental illnesses, increased SUD and hospital visits. The review has tried to link all these aspects and their role in mental health and substance use leading to hospital visits. The extreme environmental weather temperature exposure -related disorders burden the already strained healthcare facilities especially in developing countries. It also highlights that policy makers need to incorporate climate change in healthcare programs.

## 9 Strategies for prevention of extreme environmental weather temperature exposure -related disorders and healthcare system preparedness during extreme weather events

Climate change and exposures to extreme environmental temperatures is increasingly global challenges, and greatly affecting various population health often leading to ill-health (WHO, 2013, 2021, 2024; ILO, 2019; Hayes and Poland, 2018). Chronic stress due to exposure to these extreme temperatures affects mental health leading to various mental illnesses, dehydration and heat stroke, substance use and abuse, substance poisoning such as opioid and alcohol (Table 1) and even deaths (WHO, 2013, 2021, 2024; ILO, 2019; Hayes and Poland, 2018). All these cases contribute to increased hospital visits during these extreme weather temperatures. The vulnerable population is commonly affected, especially in poor nations. Whereas, in some developed countries, intervention measures against extreme weather temperatures have been put in place including providing public cooling centers in parks, museums, swimming pools; construct cool roofs, massive tree-planting initiatives especially in cities and many other measures, in low - and middle income countries (LMIC), limited or no intervention measures have been put in place to combat the extreme weather temperature challenges on health of the population in such countries. Some countries have further incorporated extreme weather temperatures in their healthcare systems though in many LMIC, this remains a challenge and often not considered as a public health challenge (WHO, 2013, 2021, 2024; ILO, 2019; Hayes and Poland, 2018). However, to address the heat stress and its associated health challenges especially mental health, the international Labor organization (ILO) and World Health Organization (WHO) and many others have provided guidance on protection of workers against extreme weather conditions especially among the vulnerable population (WHO, 2013, 2021, 2024; ILO, 2019; Hayes and Poland, 2018). These measures include instituting early warning systems that alert residents on such impending extreme weather temperatures, develop policies and enforce rules that protect workers, going green, reduce carbon gas emission and develop technologies that protect population from extreme heat exposures (WHO, 2013, 2021, 2024; ILO, 2019; Hayes and Poland, 2018). However, in many countries, especially in LMIC, these strategies have not been realized to address the challenge of extreme weather temperatures and health.

## 10 Conclusion

Prolonged exposure to extreme weather conditions, including both cold and heat, especially among the vulnerable groups such as individuals with existing mental disorders, chronic diseases, those on certain psychiatric medications, children, homeless, individuals with on shelter, older individuals, fast responders to disasters, pregnant women, outdoor workers such as construction workers, open market workers, manual workers on farms, casual laborers fisheries, low-income populations and many others; causes stress that deregulates the body’s thermoregulatory system, and the various brain neurotransmitter pathways including noradrenergic, serotonergic and dopaminergic systems with pathophysiological effects often leading to ill-health such as mental illnesses. Further, this triggers alcohol and substance use and abuse as a coping mechanism, or exacerbates their use, leading to SUD, drug intoxication, and dehydration thus leading to increased hospital visits and some cases of deaths. In addition, these extreme heat weather exposure, and their associated mental illnesses and SUD strains the already affected healthcare facilities, especially in poor nations globally.

Extreme weather temperatures drive chronic stress, ill-health and mental illnesses development, and as a way to cope with these challenges, affected individuals use alcohol and other substances, leading to SUD, intoxication, and increased hospital visits. Therefore, extreme environmental heat stress as a risk factor to mental illnesses, and alcohol and other substance abuse, and addiction pose a public health challenge, especially to the already strained healthcare facilities in low-middle-income countries for which awareness of this challenge needs to be addressed in communities as well as the development of appropriate intervention mechanisms. Therefore, nations worldwide need to incorporated extreme weather temperatures due to climate change as recommended by World Health Organization (WHO, 2013, 2021, 2024, 2025a; Dodman et al., 2023), and International Labor Organization (ILO, 2019) to promote mental health.
